# High prevalence of natural infection by the ruminant blood fluke *Schistosoma spindale* in the intermediate snail host *Indoplanorbis exustus* in Uttaradit, Northern Thailand

**DOI:** 10.14202/vetworld.2024.413-420

**Published:** 2024-02-20

**Authors:** Chorpaka Phuangsri, Ornampai Japa

**Affiliations:** 1 Division of Microbiology and Parasitology, School of Medical Sciences, University of Phayao, Phayao, Thailand; 2 Scientific Instrument and Product Standard Quality Inspection Center, University of Phayao, Phayao, Thailand

**Keywords:** *Artyfechinostomum malayanum*, cercaria, cercarial dermatitis, *Indoplanorbis exustus*, Planorbid snails, *Schistosoma spindale*

## Abstract

**Background and Aim:**

Freshwater snails belonging to the family *Planorbidae* serve as the first intermediate hosts of many species of important parasitic flukes of animals and humans. Information regarding the occurrence of planorbid snail larval trematode infection is limited in Northern Thailand. Thus, this study aimed to estimate the prevalence of larval trematode infection of the freshwater snail *Indoplanorbis exustus* in Uttaradit, Thailand, and to identify trematode species based on their morphological and molecular characteristics.

**Materials and Methods:**

Planorbid snail specimens were collected from a water reservoir in Uttaradit, Thailand, from June to August 2023. Snails were assessed for larval trematode infection through cercarial shedding and crushing methods. The released cercariae were preliminarily identified on the basis of their morphological characteristics. In addition, species identification of the detected cercariae was conducted using 28S ribosomal RNA and cytochrome c oxidase subunit 1 gene sequence analyses.

**Results:**

The overall prevalence of cercarial infection was 61.5% (107/174) in planorbid snails in Uttaradit province. Two species of cercarial trematodes, *Schistosoma spindale* (106/174, 60.9%) and *Artyfechinostomum malayanum* (1/174, 0.6%), were identified using morphological and molecular analyses, of which *S. spindale* was the most abundant species. Our studied snails did not have mixed infection with more than two cercarial species.

**Conclusion:**

Our findings reveal a remarkably high prevalence of *S. spindale* cercariae infecting planorbid snails in Uttaradit, indicating that humans and animals across the study area are at risk of infection. Our data may contribute to the development of effective strategies to control this zoonotic infectious disease.

## Introduction

The life cycle of the trematode is complex, requiring at least two hosts: An intermediate host and a definitive host. Within this cycle, freshwater snails serve as the primary intermediate hosts and play a critical role in initiating the development of parasitic larval stages, including sporocysts, rediae, and cercariae [1, 2]. The cercariae are the last larval stage that emerges from an infected snail host. They can either become immediately infectious to specific mammalian hosts or encyst as metacercariae in a suitable second intermediate host [2–4].

Many freshwater snails play a crucial role as intermediate hosts for a variety of parasites. As a result, the presence and distribution of intermediate snail hosts have significantly contributed to the widespread occurrence of various trematode-related parasitic diseases. Freshwater gastropods belonging to the family *Planorbidae* are air-breathing, freshwater mollusk species. *Indoplanorbis exustus* (Deshayes, 1834) is a common snail found throughout Southeast Asia, India, and Arabia [5]. *I. exustus* serves as an intermediate host for various parasites of medical and veterinary significance. Among the trematode parasites hosted by *I. exustus*, ruminant schistosomes are economically important trematodes that globally affect ruminants [6, 7]. Furthermore, this snail has been associated with human cercarial dermatitis outbreaks in India, Malaysia, Lao PDR, and Thailand [8–11].

Epidemiological data on cercarial trematode species infecting snails have demonstrated the transmission of trematodes into the environment from the definitive host. Understanding cercarial infection in snails is necessary to estimate the risk of trematode infectious diseases in both animals and humans in the endemic area. These data may contribute to the use of effective prevention measures and control of trematode infections in the future [12].

While morphological characteristics can be used to classify trematode cercariae and potentially group larval flukes at the family level, certain morphotypes, such as furcocercous cercariae, are found across several families, including *Diplostomidae*, *Sanguinicolidae*, *Strigeidae*, *Clinostomidae*, and *Schistosomatidae* [13]. Molecular approaches are promising for precisely identifying the trematode cercariae. Sequence analysis of 28S ribosomal RNA gene (28S rDNA) and cytochrome c oxidase subunit 1 (*COX1)* mitochondrial DNA is generally accepted as a reliable method for accurate identification of most trematode species [14–17].

Freshwater snails belonging to the family *Planorbidae* act as the first intermediate host of many species of important parasitic flukes of animals and humans [18, 19]. Information regarding the occurrence of planorbid snail trematode larval infection is limited in Northern Thailand [20]. Thus, this study aimed to estimate the prevalence of larval trematode infection of the freshwater snail *I. exustus* in Uttaradit, Thailand, and to identify trematode species based on their morphological and molecular characteristics.

## Materials and Methods

### Ethical approval

The Institutional Animal Care and Use Committee of the University of Phayao, Thailand, reviewed and approved the study protocol (Reference No. 1-020-66).

### Study period and location

Snail surveys and collection were conducted monthly from June to August 2023. Planorbid snails were randomly collected from a water reservoir in Phichai District, Uttaradit province (17°10’58.6”N, 100°02’33.8”E), Thailand, using a scoop net based on the counts per unit of time method of Olivier and Schneiderman [21]. All laboratory investigations were conducted at the School of Medical Sciences and the Scientific Instrument and Product Standard Quality Inspection Center, University of Phayao, Phayao, Thailand.

### Study design and study population

This study was a cross-sectional descriptive study. Only planorbid snails were randomly sampled to investigate parasite infection. A total of 174 snail samples were collected for this study.

### Planorbid snail identification

Collected snails were cleaned with dechlorinated water and transferred to the laboratory. Planorbid snails were primarily identified based on shell morphology, according to the taxonomic key of Brandt [22]. Snail size was measured based on a minimum of 20 randomly selected snail samples. Snails were measured using a Vernier caliper with a precision of 0.01 mm and reported as mean ± standard deviation (SD). In addition, the identification of representative planorbid snails was further confirmed molecularly by polymerase chain reaction (PCR) and sequencing of the internal transcribed spacer region 2 (ITS2) using the primers and protocols described by Bargues *et al*. [23].

### Detection of larval trematode infection

Planorbid snails were separated into clear plastic cups filled with 15–20 mL of dechlorinated water. The examined snails were assessed for trematode infection using the cercarial shedding method, in which they were initially stimulated by natural light for 3 h and then exposed to artificial light overnight. The emergence of trematode cercariae was observed using a stereomicroscope (Olympus, Japan). Discharged cercariae were collected and preserved in 70% ethanol and absolute ethanol for subsequent morphological and molecular studies. Snails negative for cercarial emergence were crushed and examined under a stereomicroscope to verify the presence of trematode larvae.

### Identification of the cercariae

The released cercariae were initially classified based on their morphological characteristics throughlight microscopy (Olympus) according to the standard identification key described by Schell [24]. In addition, ethanol-fixed cercariae were mounted with glycerol. Fresh and glycerol-mounted cercarial specimens were morphologically identified and photographed using a light microscope and confocal microscope ZEISS LSM900 (Carl Zeiss, Germany). At least 20 preserved cercariae from each morphological type were measured using a micrometer to determine the range, average, and SD, presented in micrometers (mm).

### Genomic DNA extraction

Genomic DNA was purified from snails and cercariae using a genomic DNA preparation kit (BioFact, Korea) according to the manufacturer’s protocol for animal tissue. Briefly, the tissues were homogenized in 200 µL of GD1 buffer containing proteinase K (20 mg/mL) and RNase A (4 mg/mL), followed by incubation at 56°C for 10 min. Next, 200 µL of GD2 buffer was added, and the mixture was incubated at 70°C for 10 min and centrifuged at 13,000× *g* for 5 min. After centrifugation, the clear supernatant containing DNA was transferred to a new tube, which was then combined with 200 µL of GB buffer. The mixture was carefully loaded into a spin column pre-equilibrated with Help B buffer and centrifuged for 1 min. The spin column was washed twice with 80% alcohol washing buffer. DNA was eluted using 100 µL of elution buffer. The purified genomic DNA was stored at −20°C until use.

### Molecular analysis of planorbid snails and trematode cercariae

Snail ITS2 fragment was amplified using snail-specific primers following the protocol established by Bargues *et al*. [23]. Two sets of primers were employed to amplify the 28S rDNA and *COX1* regions, as specified by Sandoval *et al*. [25] and Lockyer *et al*. [26], respectively, for analyses of emerging cercariae.

PCR was conducted using a 50 μL reaction volume and GoTaq Flexi DNA Polymerase (Promega, UK) consisting of 200 µM dNTP mix, 1.5 mM MgCl_2_, 10 pmol of each primer, 1.25 U of Taq DNA polymerase, and 1 µL of extracted DNA template. Amplification was performed using a GeneAmp PCR System 9700 (Applied Biosystems, USA). The primer sequences and PCR conditions used in this study are described in Table-1 [23, 25, 26].

**Table-1:** Sequences of primers and PCR conditions applied for the molecular analysis of both snail and trematode cercariae.

Gene	Primer	Sequences (5×–3×)	PCR condition	Reference
Snail ITS2	LT1 ITS2	TCGTCTGTGTGAGGGTCG TTCTATGCTTAAATTCAGGGG	94°C for 5 min 35 cycles of 94°C for 30s, 50°C for 30s, 72°C for 30s 72°C for 5 min	[23]
28S rDNA	CF2 CR2	GTTTGTGAATGCAGCCCAAAGT TCGCCCCTATACTCACGTTAT	94°C for 5 min 35 cycles of 94°C for 30s, 62°C for 30s, 72°C for 1 min 72°C for 10 min	[25]
*COX*1	CoxF CoxR	TCTTTRGATCATAAGCG TAATGCATMGGAAAAAAACA	94°C for 5 min 35 cycles of 94°C for 30s, 52°C for 30s, 72°C for 1.5 min 72°C for 10 min	[26]

PCR=Polymerase chain reaction, ITS2=Internal transcribed spacer region 2, *COX1*=Cytochrome c oxidase subunit 1

Amplified PCR products were analyzed using 1.5% agarose gel electrophoresis in tris, acetate, and EDTA. The agarose gel was pre-stained with Nancy-520 (Sigma-Aldrich, UK). PCR fragments of the positive samples were randomly selected for sequencing. DNA was sequenced in both directions using forward and reverse primers. Sequencing was performed using the U2Bio sequencing service (Korea).

### Sequence analyses

Sequences of the amplified fragments from snail ITS2 and parasite 28S rDNA and *COX1* were edited and assembled manually. The nucleotide sequence was subjected to an initial analysis using the standard nucleotide Basic Local Alignment Search Tool (BLAST) available in the National Center for Biotechnology Information database (http://blast.ncbi.nlm.nih.gov/). *COX1* sequences of cercariae were also subjected to BLAST search against the sequences recorded in the barcode of life data system.

The phylogenetic relationships of emerging cercariae were determined by the construction of a phylogenetic tree of 28S rDNA and *COX1*. The phylogenetic tree was constructed using the nucleotide sequences obtained, including the reference sequences. Multiple sequence alignment for each dataset was conducted using the Clustal Omega tool embedded in SeaView software version 5.0.5 (https://doua.prabi.fr/software/seaview) [27] to remove gaps or missing data. The maximum likelihood phylogenetic tree was generated using the IQ-TREE web server available at http://iqtree.cibiv.univie.ac.at/ [28]. Substitution model selection for each dataset was determined using model finder in IQ-TREE [29], and bootstrap analysis was conducted with 10,000 replicates. Nucleotide sequences of *Angiostrongylus cantonensis* with accession numbers AY292792.1 and NC_013065.1 were included as outgroups for the 28S rDNA and *COX1* phylogenetic analyses. Final trees were visualized using FigTree software version 1.4.0 (University of Edinburgh, UK) (http://tree.bio.ed.ac.uk/software/figtree/).

### Nucleotide sequence accession numbers

Snail ITS2 sequences obtained in this study were deposited in the GenBank database with the following accession numbers: OR604548-OR604554. Additionally, the nucleotide sequences of the emerging cercariae from this study were registered to the GenBank database under the accession numbers OR602911-OR602922 for 28S rDNA and OR602925-OR602933 for *COX1*.

### Statistical analysis

The prevalence of cercarial infection in the planorbid snails was calculated by dividing the number of infected snails by the total number of examined snails and presenting the result as a percentage.

## Results

### Studied planorbid snails

The collected planorbid snail shell was discoidal with a gradually increasing number of whorls. There were four to seven convex whorls in this study. The height of each whorl was greater than its width. The whorl of the body was enlarged, and the aperture was broadened and ear-shaped. The shell was flattened concavely. The shell color was greenish to grayish brown. The average size of the examined snails was 6.82 ± 0.05 × 1.25 ± 0.06 × 0.58 ± 0.05 mm.

Sequence analysis of snail ITS2 based on 480-bp amplicon showed 100% similarity to *I. exustus* isolated in Thailand (MH037126-7), Nigeria (MH037115.1, MH037106), Vietnam (MH037128.1), Oman (MH037124-5.1), Malaysia (MH037122-3), and France (MH037119-20).

### Prevalence of cercarial infection in *I. exustus* snails

During the survey, 107 snails were found to be infected with trematode cercariae using shedding and crushing methods. The overall prevalence of cercarial infection was 61.5% (107/174) among planorbid snails. Two morphotypes of trematode cercariae were recovered from larval fluke-infected snails: Furcocercous cercaria and Echinostome cercaria. In total, 106 snails (60.9%) were infected with furcocercous cercaria, while only one snail (0.6%) was infected with echinostome cercaria. There were no mixed infections of more than one cercarial type.

The morphological descriptions of the detected cercarial types are given below.

### Furcocercous cercaria

**Figure-1:**
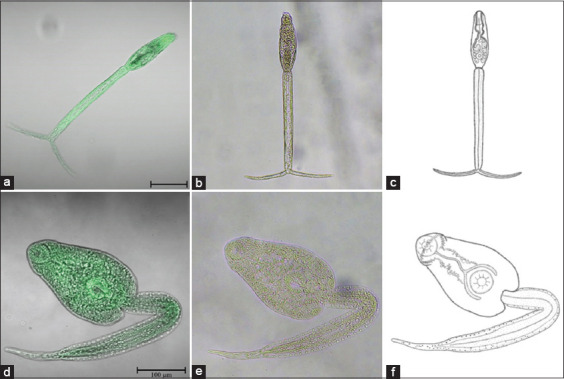
Morphology of two cercarial types from *Indoplanorbis exustus* snails. (a) Confocal microscopic image of furcocercous cercaria. (b) Light microscopic image of furcocercous cercaria. (c) Illustration of furcocercous cercaria. (d) Confocal microscopic image of echinostome cercaria. (e) Light microscopic image of furcocercous cercaria. (f) Illustration of echinostome cercaria. Scale bar: 100 μm.

The cercarial body was cylindrical, elongated, and covered with many spines. The average size of the cercarial body was 46.88 ± 6.38 × 161.38 ± 14.01 μm. The cephalic organ was clearly visible, but the pharynx and eye spots were not detected. The muscular oral sucker was large and situated anteriorly. The ventral sucker was smaller than the oral sucker and was situated closer to the posterior part of the body. The cercarial tail was slender and longer than the body, with an average tail size of 27.63 ± 4.40 × 279.00 ± 10.71 μm. The tail end was divided into bifurcations shorter than the tail stem, with an average size of 16.75 ± 5.68 × 122.60 ± 9.60 μm (Figures 1a–c).

### Echinostome cercaria

The body was pear shaped and elongated. The rounded oral sucker was located at the end of the body. The head was armed with 45–47 well-developed collar spines arranged in a single row. The ventral sucker was approximately the same size as the oral sucker, and it was located toward the posterior of the body. The esophagus was long and separated into bifurcated intestines extending to the ventral sucker. Numerous refractive granules within the main collecting tube were located on both sides of the cercarial body between the pharynx and the ventral sucker. The tail was cylindrical and longer than the body. The average size of the cercarial body was 127.50 ± 18.03 × 287.00 ± 32.94 μm, and the average size of the tail stem was 43.00 ± 7.33 × 414.00 ± 49.35 μm (Figures 1d–f).

### Sequence analysis of cercariae

PCR amplification of 28S rDNA resulted in a single amplicon of approximately 924-bp for furcocercous cercariae, whereas echinostome cercaria failed to generate a PCR product. Amplification of the 28S rDNA of furcocercous cercariae yielded consistent results for all snails positive. From the furcocercous morphological group, we selected 12 positive PCR fragments for subsequent nucleotide sequence analysis. The 28S rDNA sequences of our furcocercous cercariae had a similarity ranging from 99.13% to 100%. All 28S rDNA sequences obtained from the furcocercous cercariae showed 99.24%–100% similarity with the recorded sequences of *Schistosoma spindale* isolated from rodents in Sri Lanka (AY157257.1, Z46505.1), cattle in Bangladesh (LC224106), and snails in Nepal (KR423857.1, KR423860-2).

Amplification of the *COX1* region yielded an almost 1.2-kb fragment for both furcocercous and echinostome cercariae. *COX1* sequences of representative furcocercous cercariae were 1181-bp in length. Sequence similarity among the representatives was 99.49%–100%. Analysis of the *COX1* sequence for furcocercous cercariae indicated the highest similarity (>92%) with *S. spindale* from cattle in Bangladesh (MN637820.1) and snails in Nepal (KR607225.1). In contrast, the *COX1* sequence of the echinostome cercaria was 1192-bp and displayed the highest homology, 99.92%, to *Artyfechinostomum malayanum* (NC_065766.1).

### Phylogenetic relationships

**Figure-2:**
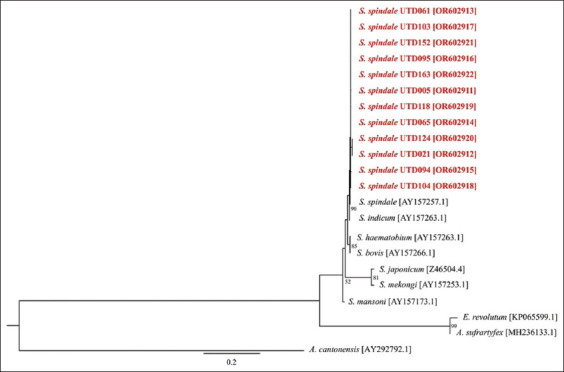
Phylogenetic relationships of the emerging cercariae from *Indoplanorbis exustus* snails and reference strains based on nucleotide sequences of 28S ribosomal DNA. Numbers at the nodes represent the bootstrap values derived from 10,000 replicates, and only values exceeding 50% are indicated. Sequences obtained from this study are presented in bold red font. Accession numbers for the sequences are enclosed in brackets. *Angiostrongylus cantonensis* (AY292792.1) was included as outgroups.

The 28S rDNA and *COX1* sequences of our cercarial specimens, along with those of known cercarial species, were used to construct a phylogeny for further analysis. The 28S rDNA phylogenetic tree revealed that all furcocercous cercariae were clustered into a single clade with *S. spindale*, which was strongly supported by the maximum bootstrap value of 90%. In addition, they were placed in a sister group containing *S. indicum* (Figure-2).

**Figure-3:**
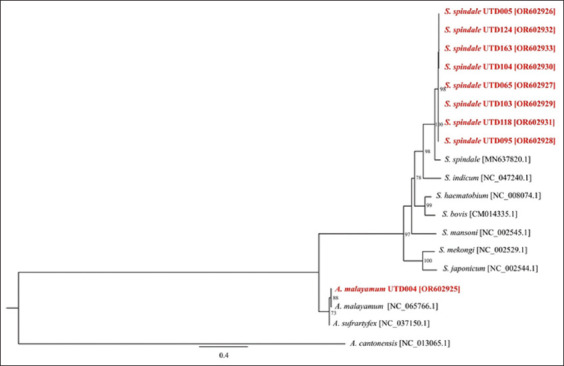
Phylogenetic relationships of the emerging cercariae from *Indoplanorbis exustus* snails and reference strains based on nucleotide sequences of cytochrome c oxidase subunit 1. Numbers at the nodes represent the bootstrap values derived from 10,000 replicates, and only values exceeding 50% are indicated. Sequences obtained from this study are presented in bold red font. Accession numbers for the sequences are enclosed in brackets. *Angiostrongylus cantonensis* (NC_013065.1) was included as outgroups.

The phylogenetic analysis of *COX1* exhibited a pattern consistent with that of 28S rDNA, in which all representative sequences of furcocercous cercariae clustered together with members of the genus *Schistosoma*. These sequences were most closely related to *S. spindale*. The echinostome cercaria formed a separate clade with other members of the genus *Artyfechinostomum*, strongly supported by a bootstrap value of 73% (Figure-3).

## Discussion

In this study, the overall prevalence of *I. exustus* trematode infections from Uttaradit, Thailand, as determined using shedding and crushing methods, was 61.5% (107/174). We observed two morphological types of trematode cercariae: Furcocercous and Echinostome. Our observed rate of cercarial infection in *I. exustus* was higher than that previously recorded in Thailand (2.05%–5.5%) [30, 31] and neighboring countries such as Malaysia (1.7%) [32] and Vietnam (38.5%) [33]. The diversity of trematode larvae in *I. exustus* was quantified. Anucherngchai *et al*. [30] observed monostome and echinostome cercariae of *I. exustus* in the Chao-Phraya Basin in Central Thailand. A preliminary screening of freshwater snails collected in Phitsanulok province by Ardpairin *et al*. [20] indicated the presence of amphistome cercaria in only one individual of *I. exustus* among the three investigated snails. In a recent study, Krailas *et al*. [31] reported that *I. exustus* from Chana, Southern Thailand, were infected with three types of cercariae: *S. indicum*, *Echinostoma spiniferum*, and *Clinostomum giganticum*.

Our analysis of cercarial morphology yielded results consistent with those previously reported for *Schistosoma* cercariae and echinostome cercariae. However, the identification of cercaria species on the basis of morphological features is a challenge that requires taxonomist expertise. Moreover, conventional microscopy cannot easily differentiate cercariae in areas where human, livestock, and wildlife cercariae coexist [34, 35]. We identified two species of trematode cercariae: *S. spindale* (60.9%, 106/174) and *A. malayanum* (0.6%, 1/174), using both morphological and molecular characteristics.

*S. spindale* is a major parasite responsible for visceral schistosomiasis in ruminants, a widespread disease across diverse Asian countries, including India, Bangladesh, Sri Lanka, Malaysia, Vietnam, Lao PDR, Indonesia, and Thailand [36]. Infections caused by *S. spindale* in livestock lead to severe health issues, imposing significant economic burdens through mortality, reduced fertility, impaired growth, reduced productivity, and increased susceptibility to further infection [37].

*S. spindale* cercariae are also known to trigger human cercarial dermatitis, an inflammatory skin reaction that occurs upon contact with water contaminated by blood fluke cercariae. Although this condition can potentially be caused by several species of animal blood flukes [38], *S. spindale* is notably one of the frequently reported sources of human cercarial dermatitis in Thailand and India [10, 39]. Kullavanijaya and Wongwaisayawan [10] documented an outbreak of cercarial dermatitis among farmers in Chaiya, Surat Thani province, Southern Thailand that was attributed to *S. spindale* cercariae. Similarly, cases of cercarial dermatitis originating from *S. spindale* cercariae have been reported in other countries, such as Malaysia and India [40, 41].

The life cycle of *S. spindale* typically involves ruminants as definitive hosts and freshwater gastropods as intermediate hosts. The presence of *S. spindale* cercariae emerging from *I. exustus* snails indicates the infection of definitive hosts with *S. spindale* in that region, indicating the active circulation of the parasite. The high *S. spindale* infection rate observed in the snails in this study could be linked to a corresponding high *S. spindale* infection rate in the ruminant definitive hosts within the area studied. Although ruminants are the primary definitive hosts of *S. spindale* in endemic areas, other wild animals, such as rodents, have also been identified as reservoirs for the parasite. The role of rodents as reservoirs has been extensively studied across various parts of the world [42–44]. In Southeast Asian countries such as Malaysia, the occurrence of *S. spindale* adults has been reported among natural reservoir hosts, including *Bandicota indica* (38.3%), *Rattus argentiventer* (14.3%), *R. rattus diardii* (1.3%), and *Rattus tiomanicus jalorensis* (1.1%) [6]. Bunnag *et al*. (1983) investigated the prevalence of *S. incognitum* in wild rodents from Phitsanulok and Phichit, Thailand, and reported a high prevalence of 41.7% [45].

Based on these observations, we hypothesize that ruminant hosts and natural reservoir hosts, such as rodents, are key sources that facilitate initial and ongoing parasite transmission within this region. The interaction between infection rates in snails and the presence of definitive and reservoir hosts highlights the potential influence of local environment on the distribution of *S. spindale*. This dynamic situation increases the likelihood of encountering infective cercariae. Consequently, animals and humans are exposed to infective cercariae in contaminated water sources in the region.

Studies focused on *I. exustus* snails in Thailand have consistently reported echinostome cercaria infections, including those by Anucherngchai *et al*. [30] and Krailas *et al*. [31]. The observed echinostome cercaria was identified as *A. malayanum* through molecular analyses of *COX1* sequences and morphological characteristics. *A. malayanum* was initially considered a synonym of *E. malayanum* [46, 47], a zoonotic intestinal fluke within the family *Echinostomatidae*. This species affects a wide range of hosts, including birds, reptiles, and various mammals, including humans [46]. Humans and animals can become infected through the ingestion of raw or undercooked secondary intermediate hosts, such as freshwater snails, tadpoles, and other aquatic organisms, which serve as carriers of metacercariae [48]. Infection by this parasite can result in severe epigastric or abdominal pain along with symptoms such as diarrhea, fatigue, malnutrition, ulceration, and bleeding [49]. Human infections have been reported across several South and Southeast Asian countries, including India, Malaysia, Indonesia, Philippines, Lao PDR, and Thailand [50–54]. Although the infection rate of *A. malayanum* was notably low in the studied snails, our findings underscore the presence of this zoonotic parasite in the area, highlighting the necessity for continuous monitoring and research attention.

## Conclusion

This study presented preliminary data on zoonotic larval trematode infection distribution in intermediate snail hosts in Uttaradit, Thailand. Our findings indicated a remarkably high prevalence of *S. spindale* cercaria infection among planorbid snails, with a correspondingly low rate of *A. malayanum* cercaria infection. The presence of ruminant schistosome *S. spindale* not only raises veterinary concerns but also has zoonotic implications for public health. The data obtained in this study could serve as foundational information regarding the occurrence, distribution, and genetic characteristics of specific parasites, particularly those infecting snails. This basic information is crucial for understanding and addressing infectious trematode diseases. However, further investigations are necessary to better understand the distributions, implications, and potential contributions of other related hosts to the circulation of *S. spindale*, which is essential for creating sustainable control measures to effectively prevent and manage *S. spindale* infection.

## Authors’ Contributions

OJ: Conceived and designed the study, carried out molecular characterization, sequencing, and bioinformatics analyses and drafted the manuscript. OJ and CP: Surveyed and collected snail specimens, performed parasite detection, identification and imaging, collected, analyzed, and interpreted data, and revised the manuscript. All authors have read, reviewed, and approved the final manuscript.
